# Disability inclusion in maternal health services in Mozambique: a mixed-methods analysis of barriers experienced by women with disabilities

**DOI:** 10.1186/s12889-026-28474-y

**Published:** 2026-07-10

**Authors:** Dulce Osório, Miguel Osório, Edy Nacarapa, Zuana Cossa, Percina Machava, Marta Nhamuanzo, Lídia Sanduane, Herval de Jesus, Amâncio Nhangave, Eden Mucache, Isabelle Munyangaju

**Affiliations:** 1 Tinpswalo Research Association to Fight AIDS and TB, Chókwè, Mozambique; 2 School of Population Health, University of New South Wales (UNSW), Sydney, Australia; 3 Frontline AIDS, Maputo, Mozambique; 4 Department of Public Health, Ministry of Health, Maputo, Mozambique; 5 Department of Pediatrics, Maputo Central Hospital, Maputo, Mozambique; 6 Internal Medicine Department, Alicante General University Hospital, Alicante, Spain; 7 Internal Medicine Department, University Miguel Hernandez de Elche (UMH), Alicante, Spain; 8 Gaza Provincial Research Nucleus, Provincial Health Directorate, Xai-Xai, Mozambique; 9 Mozambican Association for Health and Environment (AMOSA), Maputo, Mozambique; 10 School of Public Health, The University of Sydney, Sydney, Australia; 11 Pediatric Research Unit, Faculty of Medicine, Eduardo Mondlane University, Maputo, Mozambique

**Keywords:** Maternal health, Women with disabilities, Mozambique, Health equity, Inclusion

## Abstract

**Background:**

In Sub-Saharan Africa, maternal health remains a significant public health challenge, and women with disabilities (WWD) experience profound inequities in access to essential healthcare services. In Mozambique, although national strategies promote disability inclusion, systemic and structural barriers continue to undermine equitable access to maternal health services. This study explores the intersection of maternal health and disability inclusion in Mozambique, identifying barriers faced by women with disabilities and opportunities to improve service accessibility and quality.

**Methods:**

A mixed-methods design was employed, integrating a desk review with stakeholder consultations and an interactive workshop. The desk review examined national policies and relevant literature on maternal health and disability inclusion in Mozambique. Stakeholder consultations were conducted via surveys across multiple regions, engaging WWD, healthcare providers, policymakers, and disability advocates. Key findings were validated and refined through participatory workshops involving diverse stakeholders. Quantitative data were analysed descriptively, while qualitative responses were thematically analysed.

**Results:**

The study found that women with disabilities in Mozambique face multifaceted barriers to maternal health, including inaccessible infrastructure, lack of adapted medical equipment, financial constraints, inadequate provider training, communication gaps and stigmatizing attitudes. Among 81 surveyed participants, over 80% reported physical and communication barriers as major obstacles, while 62% reported attitudinal barriers. Although some participants noted the existence of policies such as the National Action Plan for Disability and the Strategy for People with Disabilities in Public Services, their implementation was widely perceived as ineffective or insufficient in improving maternal health service accessibility for women with disabilities.

**Conclusion:**

Disability inclusion within Mozambique’s maternal health system remains limited, with policy commitments insufficiently translated into practice. Achieving equitable and inclusive maternal healthcare for women with disabilities in Mozambique requires sustained investment in accessible infrastructure, provider training, community engagement, and effective accountability mechanisms. Addressing these systemic barriers is critical to improving maternal health outcomes and advancing health equity for all women in Mozambique.

**Supplementary Information:**

The online version contains supplementary material available at https://doi.org/10.1186/s12889-026-28474-y.

## Introduction

Maternal health, defined by the World Health Organization (WHO) as the well-being of women during pregnancy, childbirth, and the postpartum period, is fundamental to the development of healthy and productive societies [1]. Key indicators for assessing maternal health include reductions in maternal mortality, access to sexual and reproductive health services, and progress toward universal health coverage [1]. Despite substantial progress in reducing maternal mortality over the past decades, globally, about 800 women die every day from preventable maternal causes, most occurring in low-income countries (LIC) [2]. While maternal health challenges affect women generally in many LIC, particularly in Sub-Saharan Africa (SSA), these challenges are more pronounced among women with disabilities (WWD), who often receive less attention in health policy and service delivery [3, 4]. 

Disability is an important public health issue globally. According to WHO, Disability is a broad term encompassing impairments, activity limitations, and participation restrictions that arise from the interaction between an individual with a health condition and their surrounding environmental factors [5]. Globally, around 1.3 billion people (16% of the global population), live with disabilities, with approximately 80% of them living in low-and middle-icome countries (LMIC) [5]. People with disabilities face higher mortality rates, poorer health outcomes, and greater functional limitations due to health inequities driven by factors such as stigma, discrimination, poverty, exclusion from education and employment, and systemic barriers within healthcare [5–7]. 

In Mozambique, around 727,620 individuals (2.7% of the population) live with disability, nearly half of whom are women [8]. Despite overall health gains over time, specific groups, including women and people with disability, continue to experience significant health disparities and poorer health outcomes. Structural factors, such as the socioeconomic and political environment, along with socioeconomic status, and living conditions, are key social determinants of health that contribute to these health disparities in Mozambique [9, 10]. 

Women with disabilities face some of the most significant health and social inequalities, particularly in sexual and reproductive health; a domain often neglected in both policy and practice. These inequities persist across the continuum of care, from family planning and antenatal care to childbirth and postpartum services [3, 11]. As a result, WWD experience disproportionately higher maternal mortality and morbidity rates, limited access to critical health information and services, and a greater incidence of pregnancy complications, preterm births, and low birth weight infants [3, 4, 11]. The pervasive misconception that WWD are not sexually active further exacerbates their exclusion, reinforcing systemic neglect and lack of tailored maternal health services [3, 12–14]. Together, these inequities underscore an urgent need to address disability inclusion as a central priority in maternal health.

This study, therefore, explores the intersection of maternal health and disability in Mozambique, identifying barriers to inclusive care and proposing actionable strategies to strengthen equity in service delivery.

## Materials and methods

### Study design

This study employed a mixed-methods approach to assess disability inclusion in maternal health services in Mozambique. The study combined three complementary components: a structured desk review of policy and literature, a stakeholder consultation conducted through a survey generating both quantitative and qualitative data, and an interactive stakeholder workshop. A convergent design was applied, whereby data from these components were collected within the same study period, analysed separately, and integrated during interpretation. This approach enabled the combination of policy-level evidence with stakeholder perspectives and lived experiences of women with disabilities, providing a comprehensive understanding of barriers to maternal health services. Findings were triangulated to enhance the depth, validity, and contextual relevance of the results. This approach aligns with established definitions of mixed-methods research, which emphasise the systematic collection, analysis, and integration of quantitative and qualitative data to provide a more comprehensive understanding of complex health issues [15]. 

### Data collection

Three complementary data collection methods were used to collect both secondary and primary data:

1. Desk review

A structured desk review was conducted to identify relevant policy documents, national guidelines, and literature on maternal health and disability inclusion in Mozambique. Searches were conducted in PubMed, Google Scholar, and Google between July and August 2024 to capture both peer-reviewed and grey literature. No date limits were imposed on the search strategy.

A combination of keywords and Boolean operators was used, including terms such as “maternal health” OR “maternal care”, OR “reproductive health” combined with “disability”, “persons with disabilities”, OR “women with disabilities”, AND “Mozambique”. Additional searches incorporated broader terms such as “health services accessibility” and “health policy” to capture relevant contextual and regional evidence. A detailed description of the search strategy is provided in Supplementary File 1. Documents were eligible if they addressed maternal health, disability inclusion, or health policy in Mozambique. Publications in English or Portuguese were included, with no time restrictions applied. Duplicates and documents not relevant to the study objectives were excluded. The search, screening, and data charting processes were conducted by two authors (D.O and I.M). Titles, abstracts, and full texts were screened for relevance against predefined inclusion criteria. Data were extracted using a structured template capturing document characteristics, key findings, and relevance to disability inclusion in maternal health. Discrepancies in screening or data extraction were discussed and resolved by consensus. Extracted data were synthesised narratively according to predefined themes, including policy frameworks, service accessibility, and disability inclusion gaps.

2. Stakeholder consultations

Stakeholder consultations were conducted between November and December 2024 using a structured questionnaire administered both online and in person. In-person data collection was limited to Maputo City due to logistical constraints, where participants could choose their preferred mode of participation, facilitating the inclusion of individuals with disabilities and those with limited access to technology. Online administration enabled inclusion of participants from other regions and those unable to attend in person. Participants were selected using purposive sampling to encourage representation across provinces, disability types, and institutional sectors. Eligible participants included WWD, healthcare providers, policymakers, and disability advocates. The survey was designed to generate exploratory and descriptive insights into stakeholder perspectives rather than statistically generalisable findings. The questionnaire was specifically developed for this study in English and Portuguese and included both closed-ended questions, generating quantitative data, and open-ended questions, generating qualitative data. These questions addressed access to maternal health services, experienced barriers, and proposed solutions for improving disability inclusion. The instrument was piloted with a small group of stakeholders to assess clarity, relevance, and comprehensibility, and minor revisions were made accordingly. The English version of the questionnaire is provided as Supplementary File 2.

3. Interactive workshop

An interactive stakeholder workshop was conducted on the 31st of January 2025 in Maputo in a hybrid format, with participants attending both in person and online. The workshop brought together policymakers, healthcare providers, WWD, and disability advocates to ensure a multi-sectoral perspective. The session lasted approximately two hours and was facilitated using participatory approaches. Discussions were audio-recorded and complemented by detailed notetaking. The workshop aimed to present and validate survey findings, explore emerging themes, and identify practical strategies to improve disability inclusion in maternal health services.

### Data analysis

Data from the desk review were synthesised using a narrative approach, grouping findings according to policy domains and thematic relevance. Quantitative survey data were entered into Microsoft Excel (Office 365, Version 2408) and analysed using descriptive statistics, including frequencies and percentages. These findings are presented to illustrate patterns in stakeholder perspectives and should be interpreted as indicative rather than representative. Qualitative data from open-ended survey responses and workshop discussions were analysed thematically using an inductive approach to identify recurrent themes related to barriers, experiences, and potential solutions. Findings from all three components were triangulated to identify consistent patterns and to validate emerging recommendations.

### Ethical considerations

This study did not involve clinical interventions. The desk review relied on publicly available documents. Participation in surveys and workshops was voluntary, with informed consent obtained from participants prior to data collection. Responses were anonymized to ensure confidentiality. Ethical approval was obtained from the Institutional Bioethics Committee for Health of Gaza (Reference No. 197/CIBS-Gaza/2024). Participant responses were anonymized, and all data were stored securely with access limited to the research team.

## Results

### 1. Desk review findings

A total of 142 records were initially identified through database and grey literature searches. After removing duplicates using EndNote, 118 records were screened. Title and abstract screening led to the exclusion of 96 records that did not meet the inclusion criteria, primarily due to lack of relevance to maternal health or disability inclusion, in the Mozambican context. Full-text assessment was conducted for the remaining 22 records, of which 11 were excluded for not sufficiently addressing the study objectives or lacking substantive content on disability or maternal health. Ultimately, 11 records were included in the review.

The desk review examined both peer-reviewed and grey literature, including policy documents and national strategies (*n* = 4), technical and institutional reports (*n* = 4), and peer-reviewed articles (*n* = 3). Policy documents included national frameworks related to disability and gender, while reports provided contextual evidence on health systems, inequalities, and sexual and reproductive health. Peer-reviewed studies contributed empirical evidence on maternal health service utilisation and access barriers among vulnerable populations.

#### Disability policies in Mozambique

Mozambique ratified the Convention on the Rights of Persons with Disabilities (CRPD) and its Optional Protocol in 2012, signalling a formal commitment to promoting and protecting the rights of persons with disabilities in line with global standards for disability rights [16]. This commitment builds on earlier national frameworks, including the Policy of Person with disability (1999), which established the initial foundation for recognising disability as a matter of public health, social inclusion, and human rights in Mozambique [17]. Within the health sector, this policy emphasises prevention, integrated care, and rehabilitation, including maternal and child health interventions aimed at reducing the incidence of disability, as well as the integration of persons with disabilities into general health services [17]. However, the policy adopts a predominantly preventive and biomedical approach, with limited attention to gender-specific needs or the rights and autonomy of women with disabilities.

Building on this foundation, Mozambique later developed more comprehensive frameworks such as the National Action Plan for the Area of Disability (*PNAD* I 2006–2010 and *PNAD* II 2012–2019) to guide the promotion of inclusion and equal opportunities for persons with disabilities [18, 19]. The PNAD II adopts a rights-based and multisectoral approach, aiming to improve access to essential services such as health, education, and social protection, and to reduce stigma and discrimination against persons with disabilities. Within the health sector, the plan emphasises improving access to general health and rehabilitation services, including physiotherapy and assistive devices, and calls for the integration of disability into broader public health policies [18]. 

In addition to broad national strategies, Mozambique has also implemented sector-specific policies, such as the Strategy for Persons with Disabilities in the Public Service (2009–2013), which focuses on promoting inclusion within public sector employment [20]. This strategy prioritises inclusive recruitment, workplace accessibility, professional development, and the reduction of institutional discrimination, reflecting a strong commitment to disability inclusion within the labour sector. It also highlights the importance of addressing physical, institutional, and attitudinal barriers, and emphasises the need for accessible infrastructure, adapted communication, and capacity building within public institutions [20]. 

Despite this progress, the national legal and policy framework continues to present significant gaps that prevent it from adequately promoting and protecting the rights of the diverse population of people with disabilities. People with disabilities face persistent barriers, including inaccessibility, limited access to information, and discriminatory practices [16]. Across the reviewed documents, no policy explicitly addresses maternal health needs of women with disabilities, and no specific provisions were identified related to inclusive reproductive or maternal health services. Similarly, no clear indicators or funding mechanisms were found to support the implementation or monitoring of disability-inclusive maternal health care. While the 1999 policy includes maternal and child health primarily within a preventive framework, and later policies such as the PNAD II promote general access to health services, these do not include explicit or targeted provisions addressing maternal health services for women with disabilities.

#### Maternal health in Mozambique

Maternal health remains a major public health challenge in Mozambique, despite gradual improvements in recent decades. The Sustainable Development Goals emphasize expanding equitable access to quality healthcare and providing integrated services as key strategies for reducing maternal mortality [18, 21]. Mozambique continues to report one of the highest maternal mortality rates in Sub-Saharan Africa, estimated at 223 deaths per 100,000 live births in 2023 [22]. While it dropped by 72% from 798 in 2000 to 223 in 2023, it remains high [23]. No disaggregated data on maternal mortality among women with disabilities are currently available in Mozambique, making it difficult to monitor equity in maternal outcomes. Available health data systems predominantly report aggregated indicators, with limited disaggregation by vulnerability characteristics, including disability [21]. 

National policy frameworks emphasise maternal health and sexual and reproductive health as key priorities for improving women’s health outcomes and reducing mortality [18]. These frameworks highlight the importance of expanding access to antenatal care, skilled birth attendance, emergency obstetric care, and family planning services as central strategies within the health sector [18]. Implementation of these priorities is structured through the expansion of primary healthcare services, strengthening of referral systems, and integration of maternal health within broader reproductive health programmes. However, the delivery of these services is influenced by health system constraints, including shortages of trained personnel, limited infrastructure, and unequal distribution of resources across regions [21]. 

A significant portion of the population, approximately 32.6%, lacks access to healthcare services, highlighting the overall difficulty in accessing maternal healthcare [16]. Evidence shows that the access and utilisation of maternal health services is significantly lower among women who are poor, live in rural areas or less empowered [21, 24]. Key barriers to accessing maternal health services include long distances to health facilities, transport costs, shortages of healthcare personnel, and limited availability of essential medicines and equipment. In addition, sociocultural factors such as gender norms, stigma, and traditional beliefs influence health-seeking behaviours and utilisation of maternal health services [21]. 

For WWD, challenges in accessing maternal health services are even greater compared to the general population. Public maternal healthcare services are generally under-resourced and poorly equipped to address the specific needs of WWD [23]. Evidence indicates that persons with disabilities face additional barriers to accessing sexual and reproductive health services, including inaccessible infrastructure, lack of adapted communication, and discriminatory attitudes within healthcare settings [23]. Health systems are reported to have limited capacity to provide inclusive services, with insufficient training of healthcare providers and lack of targeted interventions addressing the needs of persons with disabilities. Furthermore, disability is not systematically integrated into sexual and reproductive health programmes, and there is limited inclusion of persons with disabilities in policy implementation and service delivery planning [23]. 

#### Barriers to healthcare access for women with disabilities

Women with disability often encounter multiple and intersecting barriers that hinder their access to essential maternal health services, including structural, financial, and attitudinal barriers.

- **Structural barriers:** The healthcare infrastructure in Mozambique is not adequately equipped to serve the needs of WWD. A 2023 analysis revealed that health facilities are physically inaccessible, lacking necessary features such as ramps, adequately sized bathrooms, and specialized equipment designed to meet the needs of physically disabled individuals [25]. This issue is pronounced in maternal health services, where antenatal and perinatal care are often out of reach for WWD due to shortage of appropriate resources including delivery beds that can accommodate women with physical disabilities or shorter stature [26]. Additionally, crucial information regarding sexual and reproductive health, family planning, and rights remain inaccessible to women with visual, hearing, intellectual, or other disabilities, who require alternative communication methods to fully understand and utilize these services [16, 26].
- **Financial barriers:** Financial constraints represent a substantial obstacle for people with disabilities [25]. The economic burden associated with disability often includes additional costs for medical care, assistive devices, transportation, and other necessities, which can strain limited financial resources. In Mozambique, where poverty is widespread, these financial challenges are exacerbated for people with disabilities, who already face reduced earning potential due to systemic discrimination in employment and education [16]. Furthermore, health facilities are often located at long distances from communities, leading to additional transportation costs, which further limit access for WWD. Women with disabilities face even fewer employment opportunities compared to women without disabilities, intensifying their financial vulnerability and reducing their ability to afford necessary healthcare services [26].
- **Attitudinal and communication barriers:** Stigmatization and discriminatory behaviour from some healthcare professionals are common experiences for WWD in Mozambique [16]. Additionally, women with visual and hearing impairments encounter communication barriers due to the lack of sign language interpreters and alternative communication methods such as materials in Braille or easy-to-read formats of [25].

These barriers compound across the continuum of maternal care, from antenatal visits to delivery and postnatal follow-up. 

### 2. Stakeholders’ consultation and workshop

This section presents findings from stakeholder consultations, including quantitative data derived from closed-ended survey questions, presented as descriptive statistics (percentages and frequencies), and qualitative insights from open-ended survey responses and workshop discussions. Given the purposive sampling approach, these findings are intended to provide indicative insights and should not be interpreted as representative of all stakeholders in Mozambique.

#### Participant characteristics

A total of 81 stakeholders participated in the survey, representing diverse regions across the country, with the highest proportion (44%, *n* = 36) from Maputo City. Participants included public healthcare workers (40%, *n* = 32), policymakers (6%, *n* = 5), non-governmental organization representatives (37%, *n* = 30), and people with disabilities (17%, *n* = 14). The majority of participants (90%, *n* = 73) reported five or more years of experience working with women or in maternal health.

#### Perceptions of maternal health services

**Fig. 1:**
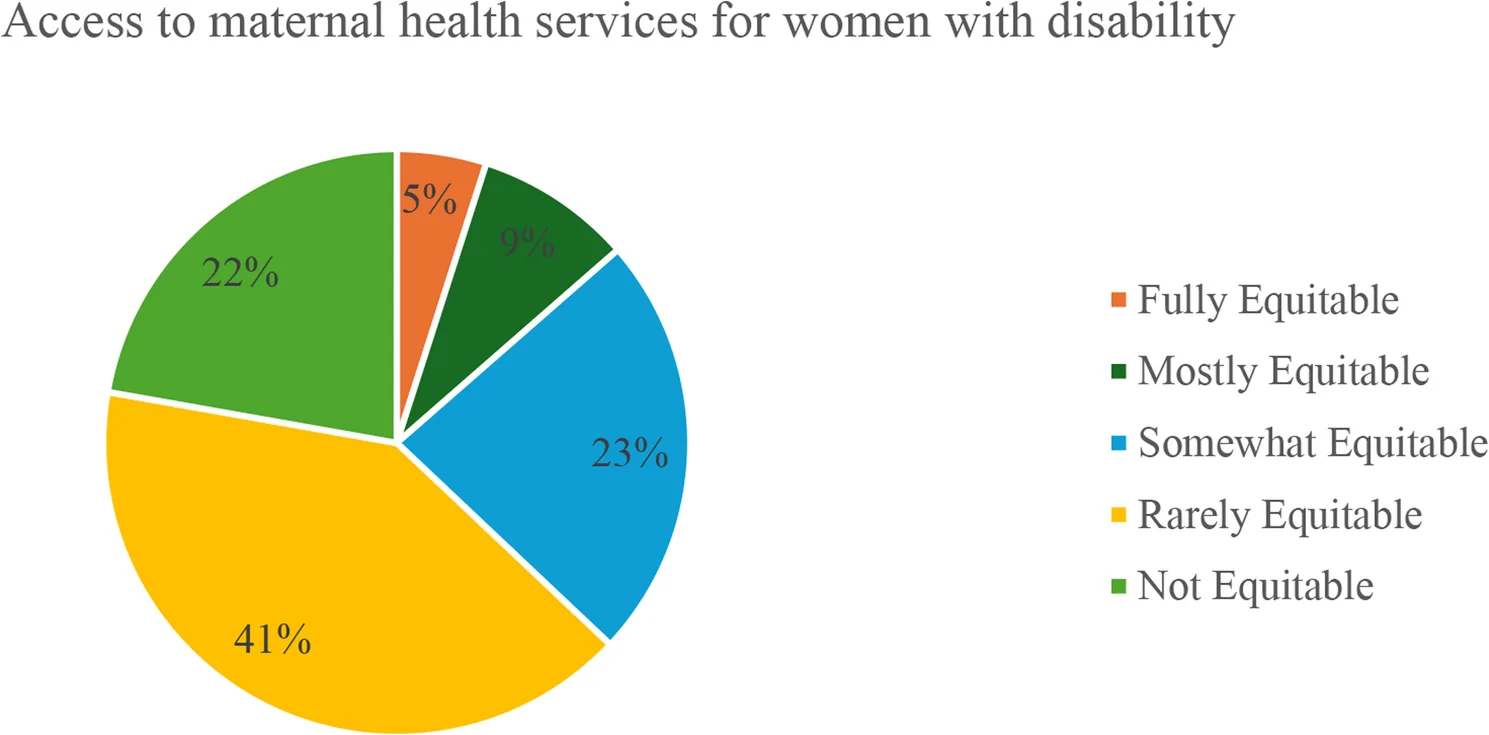
Participants responses (*N* = 81) regarding equity in access to maternal health services in Mozambique

Among respondents, 57% (*n* = 46) rated maternal health services as unsatisfactory or very unsatisfactory. With regard to equity in access to maternal healthcare for women with disabilities, 63% (*n* = 51) described services as rarely equitable or not equitable (Fig. 1). Additionally, 40% (*n* = 32) of participants reported that maternal health services do not meet the specific needs of women with disabilities at all, while 33% (*n* = 27) indicated that services only partially meet these needs. Furthermore, approximately 80% (*n* = 65) of participants reported that healthcare providers are not adequately trained to address the needs of women with disabilities.

#### Barriers and challenges to maternal health services

**Fig. 2:**
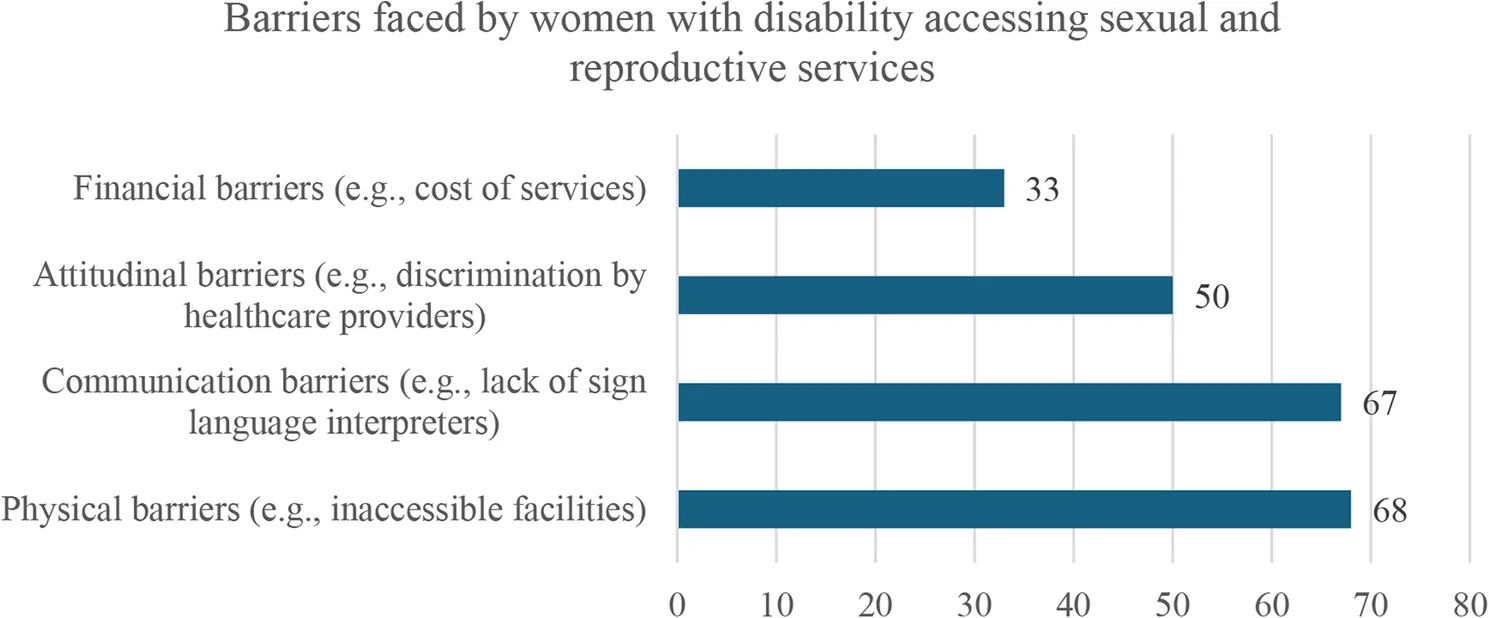
Participants responses (*N* = 81) regarding the barriers faced by WWD accessing sexual and reproductive services in Mozambique. The chart indicate that the bars reflect number of people responding

Participants identified multiple and intersecting barriers affecting access to maternal health services for WWD. Findings from the closed-ended survey questions indicate that structural and communication barriers were predominant, followed by attitudinal, and financial barriers. These patterns are illustrated in Fig. 2, which shows the distribution of reported barriers across categories.

Structural barriers were the most frequently reported, cited by 84% (*n* = 68) of respondents. These included inaccessible infrastructure such as lack of ramps, narrow doorways, non-adjustable delivery beds, and absence of accessible sanitation facilities. Qualitative findings from open-ended survey responses and workshop discussions highlighted that these barriers were particularly limiting during antenatal care and delivery.

Communication barriers were reported by 83% (*n* = 67) of participants and included the absence of sign-language interpreters and lack of accessible information formats, such as Braille or easy-to-read materials. Qualitative accounts indicated that these limitations restricted women’s ability to understand health information and engage effectively with maternal health services.

Attitudinal barriers were identified by 62% (*n* = 50) of respondents, and included experiences of stigma, discrimination, and judgmental attitudes from healthcare providers. Qualitative findings further revealed that some WWD felt their reproductive choices questioned, and that lack of privacy and respectful care negatively influenced their willingness to seek services.

Financial barriers were reported by 41% (*n* = 33) of participants and included costs related to transportation, healthcare services, and assistive devices. Qualitative data suggested that these constraints were particularly important for women with disabilities, who often face reduced economic opportunities and increased dependency.

Across all domains, qualitative findings from open-ended survey responses and workshop discussions reinforced the quantitative results, illustrating how these barriers interact and accumulate across the continuum of care, from antenatal services to delivery and postnatal follow-up. These findings are summarised in Table 1, which presents the frequency of barriers reported in the closed-ended survey alongside illustrative qualitative quotes from open-ended responses and workshop discussions.

**Table 1:** Barriers to maternal health services for women with disabilities, grouped by category in Mozambique

Category	Specific Barriers Identified	% of Participants Reporting (*N* = 81)	Illustrative Quotes
Structural/physical Barriers	Lack of ramps and accessible toilets; narrow doorways; non-adjustable delivery beds; absence of adapted equipment.	84% (*n* = 68)	“The main challenges are distance, the lack of ramps in some health facilities, and beds that are not appropriate for women with physical disabilities.” “…lack of accessibility for people with disabilities—for example, as a person with short stature, I cannot get onto the beds and there is no assistance to help me. There is a lack of ramps, inappropriate showers…”
Financial Barriers	Cost of transport to health facilities; absence of disability-friendly transport; out-of-pocket payments for services or assistive devices	41% (*n* = 31)	“The lack of transportation, such as ambulances, and in some cases the long distances to the health facility.” “Extreme poverty.”
Attitudinal	Discriminatory behaviours and stigmatizing assumptions about WWD. Many participants described negative experiences, including health workers questioning the legitimacy of WWD’s pregnancies, lack of privacy, judgmental treatment, and in some cases, obstetric violence.	62% (*n* = 50)	“There is judgment because of our disability and discrimination. People need to get it out of their heads that a woman with a disability cannot have sex and have children.” “The maternity ward has no privacy. I always have to bring a companion to the health facility, which is uncomfortable because I do not speak freely and sometimes have personal concerns.”
Communication Barriers	Absence of sign-language interpreters; lack of materials in Braille or easy-to-read formats	83% (*n* = 67)	“Lack of communication (absence of professionals who can communicate in sign language, lack of communication in Hebraic).”

Percentages reflect responses to closed-ended survey questions. Illustrative quotes are drawn from open-ended survey responses and workshop discussions

#### Policy awareness and gaps

Awareness of existing policies or programmes addressing disability inclusion was limited, with only 35% (*n* = 28) of participants reporting familiarity with such frameworks. Among those aware, the most commonly cited were the National Action Plan for Disability (*Plano Nacional da Área da Deficiência*) and the Strategy for People with Disabilities in Public Services (*Estratégia da Pessoa com Deficiência na Função Pública*). However, 42% (*n* = 12) of these respondents considered these frameworks to be ineffective or insufficient in improving accessibility of maternal health services for women with disabilities.

## Discussion

Women with disability face significant challenges accessing maternal health services, particularly in settings where health systems are already under strain. This study examined the intersection of maternal health and disability in Mozambique, a country where maternal mortality remains among the highest in SSA and where WWD are particularly underserved. Despite Mozambique’s formal commitments to disability rights through ratification of the CRPD in 2012, findings from the desk review, stakeholder consultations and workshop reveal a profound gap between policy intent and implementation. Findings from the desk review further demonstrated that, although multiple national policies and strategies address disability and gender more broadly, none explicitly incorporate provisions for disability-inclusive maternal health services, and no specific monitoring indicators or financing mechanisms were identified. Women with disability continue to experience inequitable access to maternal health services due to inaccessible infrastructure, financial constraints, communication challenges, and stigmatizing provider attitudes. These barriers occur within an already strained health system, reflecting the structural and systemic nature of exclusion.

The barriers identified in this study align with evidence from other SSA contexts, where WWD consistently report profound difficulties accessing and utilising maternity services including antenatal care, facility delivery and post-natal care [27]. Previous studies have consistently reported that visually impaired women struggle with inaccessible information and facility design, while women with hearing impairments face major communication challenges due to the absence of sign-language interpreters or adapted materials. Physically disabled women encounter facilities without ramps, small consultation rooms, and non-adjustable beds [13, 28]. These obstacles mirror those identified in Mozambique, suggesting that such barriers are systemic across the region rather than context specific.

Negative and discriminatory attitudes from healthcare providers emerged as one of the most pervasive barriers in this study. Participants described being judged or questioned about their right to motherhood. These findings are consistent with global evidence and studies from SSA showing that stigma and negative provider attitudes are major deterrents to equitable maternal health for WWD [27, 29]. Further supporting discriminatory attitudes from health workers, a study in Ghana found that some health professionals had negative perceptions questioning the need of WWD getting pregnant and maintaining it [30]. Stigma and implicit bias in provider–patient interactions undermine not only access but also the quality of care and trust in the health system. Nearly four in five stakeholders in our consultations stated that healthcare workers were not adequately trained to address the specific needs of WWD, a pattern consistent with findings across SSA [27]. These attitudes, rooted in social stigma and lack of professional training, highlight the urgent need for institutional reforms to embed disability inclusion within all levels of health-worker education.

These barriers are not only inconveniences to overcome; they represent a fundamental weakness of the healthcare system to recognize and address the diverse needs of WWD. Despite lack of data, the available evidence consistently shows that WWD face elevated risks of adverse maternal outcomes, underscoring the need for care that is not only accessible but also inclusive and responsive to their specific needs [31–33]. The pervasive nature of these challenges across the region suggests a broader systemic problem, in which healthcare infrastructure, policies, and practices have not evolved sufficiently to accommodate all individuals. This lack of inclusivity perpetuates health disparities and social inequalities, highlighting the urgent need for comprehensive reforms that prioritize accessibility, equity, and quality of care for vulnerable populations.

Although Mozambique has adopted policies such as the National Action Plan for Disability and the Strategy for People with Disabilities in Public Services, their impact remains limited. Participants in this study perceived these frameworks as largely symbolic, with insufficient enforcement, monitoring, and funding. Weak coordination between disability and maternal-health programs, poor dissemination of guidelines, and the absence of accountability mechanisms contribute to implementation failure, a problem similarly reported by policymakers in earlier analyses of women’s health programs in Mozambique [34]. These challenges mirror broader structural issues in SSA, where ambitious health policies often falter during implementation due to resource constraints, limited local ownership, and workforce shortages [35, 36]. Collectively, these findings suggest that without stronger accountability mechanisms, sustained investments, and disability-sensitive training for healthcare workers, policy commitments will remain largely symbolic, with limited impact on equitable access to maternal health services for WWD.

Applying the WHO Health Systems Building Blocks lens [37, 38], the barriers identified span multiple domains. Service delivery remains constrained by the absence of ramps, adapted delivery beds, and disability-friendly toilets. Health workforce gaps include inadequate pre-service and in-service training on disability inclusion, lack of sign-language skills, and minimal sensitivity to gender and disability rights. Health information systems do not disaggregate data by disability, preventing effective monitoring of outcomes and policy progress. Governance and leadership remain weak, with limited accountability and coordination across sectors. Addressing these deficiencies requires cross-cutting reforms that combine infrastructure investment, training, inclusive policy enforcement, and strong community partnerships.

Engaging disability associations and community health workers could help to strengthen outreach, reduce stigma, and facilitate patient feedback mechanisms. Community-based initiatives, including participatory audits of facility accessibility, have proven effective in other settings and could be adapted for Mozambique [39, 40]. Moreover, integrating disability indicators into national health-information systems would enable systematic monitoring of service access and maternal outcomes for WWD, closing the current data gap.

### Strengths and limitations

This study provides novel insights by integrating policy analysis with first-hand perspectives from WWD, healthcare providers, and policymakers, offering a comprehensive assessment of disability inclusion gaps in maternal health. A key strength of the study is its mixed-methods design, which triangulates evidence from policy review, quantitative survey’s findings, and qualitative insights from key stakeholders across multiple regions of the country. This methodological triangulation enhanced the depth and validity of the findings by capturing both documentary evidence and lived experiences. Importantly, the inclusion of WWD as active participants added essential lived perspectives that are often overlooked in maternal-health research.

Despite these strengths, several limitations must be acknowledged. First, the desk review identified a limited number of relevant documents, reflecting gaps in available evidence on the intersection between maternal health and disability in Mozambique. The existing literature primarily focused on general maternal health indicators or disability rights but rarely examining the specific challenges faced by WWD in accessing maternal healthcare. Second, the small and non-random sample of the survey limits generalizability, and reliance on self-reported perceptions may introduce recall or social-desirability bias. In addition, while this study incorporated qualitative data through open-ended survey responses and an interactive stakeholder workshop, it did not employ more in-depth qualitative methods such as individual interviews or dedicated focus group discussions (FGDs). Although the workshop provided valuable group-based insights and allowed for interactive validation of findings, more intensive qualitative approaches could have generated deeper, more nuanced understanding of lived experiences and implementation challenges. This represents an important area for future research. Finally, data collection was conducted during a period of socio-political unrest, which may have limited participation from certain regions. Despite these constraints, the use of multiple data sources and triangulation across methods strengthened the credibility and consistency of the findings providing valuable evidence in an underexplored topic and offering important insights to inform future research, policy development, and programmatic efforts to strengthen inclusive maternal health services for WWD in Mozambique.

### Implications for policy and practice

The findings of this study highlight several priority areas for strengthening disability inclusion within Mozambique’s maternal health system. Improving physical accessibility of health facilities remains fundamental, including the provision of ramps, accessible sanitation facilities, and appropriately designed delivery and examination equipment. Equally important is strengthening the capacity of healthcare providers through the integration of disability inclusion into both pre-service and in-service training, with particular emphasis on respectful care, effective communication, and awareness of the rights and specific needs of WWD.

At the policy level, there is a need to move beyond general commitments to disability inclusion towards the development and implementation of targeted strategies that explicitly address maternal health services for WWD. These efforts should be supported by robust monitoring frameworks, dedicated resources, and strengthened accountability mechanisms to ensure effective implementation. Enhancing community engagement, including partnerships with organisations of persons with disabilities, may further contribute to reducing stigma, improving trust in the health system, and increasing service uptake.

Finally, addressing financial barriers and integrating disability-disaggregated indicators into national health information systems will be critical to improving equity in access and enabling systematic monitoring of maternal health outcomes among WWD. Collectively, these actions are essential to bridge the gap between policy and practice and to advance progress towards the Sustainable Development Goals, particularly Goal 3 (health and well-being) and Goal 10 (reduced inequalities), by ensuring that maternal health systems are inclusive, equitable, and responsive to the needs of all women [41, 42]. 

Future research should prioritize generating disaggregated data on maternal health outcomes among WWD, as current national health statistics do not capture these disparities. Additionally, intervention-based studies are needed to evaluate the effectiveness of specific strategies such as provider training, infrastructural adaptations, and financial support mechanisms, in improving equitable access and outcomes for WWD.

## Conclusion

Collectively, the findings indicate that disability inclusion within Mozambique’s maternal-health system remains limited and uneven. Women with disabilities continue to face multiple and intersecting barriers to accessing maternal health services, reflecting persistent gaps between policy commitments and implementation. Addressing these challenges will require coordinated action across the health, disability, and social sectors, including efforts to strengthen accessibility, provider capacity, accountability, and disability-inclusive monitoring systems. Such actions are essential to advance equitable maternal healthcare and ensure that no woman is left behind.

## Supplementary Information

Supplementary Material 1.

Supplementary Material 2.

## Data Availability

De-identified data underlying the findings are available from the corresponding author upon reasonable request. Relevant policy documents analysed in this study are publicly available and can be accessed through the references provided.
