# Supplementary material for: Disability inclusion in maternal health services in Mozambique: a mixed-methods analysis of barriers experienced by women with disabilities
Source: BMC Public Health. 2026 Jul 10;26:2872. doi: 10.1186/s12889-026-28474-y (PMC13647631; doi:10.1186/s12889-026-28474-y)
Supplement: Supplementary file 1 [file 12889_2026_28474_MOESM1_ESM.docx]

**Supplementary Material File 1: Search Strategy**

**Search Strategy and Information Sources**

A structured desk review was conducted to identify peer-reviewed and grey literature on maternal health and disability inclusion in Mozambique.

**Data Sources**

The following sources were systematically searched:

- PubMed
- Google Scholar
- Google (for grey literature, including policy documents, reports, and institutional publications)

The search was conducted with no time restrictions applied.

**Search Strategy**

**1. PubMed Search Strategy**

The PubMed search combined Medical Subject Headings (MeSH) and free-text terms (title/abstract) to maximise sensitivity and specificity.

Search string:

( "Maternal Health"[Mesh] OR	"Maternal Welfare"[Mesh] OR	"Pregnancy"[Mesh] OR	"Reproductive Health Services"[Mesh] OR	"maternal health"[tiab] OR	"maternal care"[tiab] OR	"maternal services"[tiab] OR	"reproductive health"[tiab]) AND ("Disabled Persons"[Mesh] OR	"Disability Evaluation"[Mesh] OR	"disability"[tiab] OR	"persons with disabilities" [tiab] OR	"women with disabilities"[tiab]) AND ("Mozambique"[Mesh] OR	Mozambique [tiab])

**Expanded Searches**

To broaden the scope of evidence, additional searches were conducted using the following structured queries:

**Health Services Accessibility**

(“Health Services Accessibility"[Mesh] OR	"Delivery of Health Care"[Mesh] OR	"access to care"[tiab] OR	"health services accessibility"[tiab]) AND ("Disabled Persons"[Mesh] OR	"disability"[tiab]) AND ("Mozambique"[Mesh] OR Mozambique[tiab])

**Health Policy and Systems**

(“Health Policy"[Mesh] OR	"health systems"[tiab] OR	"health policy"[tiab]) AND ("Disabled Persons"[Mesh] OR	"disability inclusion"[tiab]) AND ( “Mozambique"[Mesh] OR Mozambique[tiab])

**2. Google Scholar and Grey Literature Search**

Search terms were adapted to suit non-indexed databases and search engines. Examples included:

“Maternal health disability Mozambique”, “women with disabilities maternal care Mozambique”, “access to health services disability Mozambique”, “health policy disability Mozambique”

Screening was conducted based on relevance, reviewing the first 100 results per search query.

**Additional Search Methods**

- Reference lists of included studies and reports were manually screened to identify additional relevant sources.
- Forward citation tracking was conducted using Google Scholar where applicable.
- Targeted searches of institutional and organisational websites (e.g., government, UN agencies, NGOs) were performed to identify additional grey literature.

**Notes**

- Search terms were adapted for each database as appropriate.
- No restrictions were applied on publication date.
- Searches were conducted in English; key documents in Portuguese were included where identified.
