# Supplementary material for: Disability inclusion in maternal health services in Mozambique: a mixed-methods analysis of barriers experienced by women with disabilities
Source: BMC Public Health. 2026 Jul 10;26:2872. doi: 10.1186/s12889-026-28474-y (PMC13647631; doi:10.1186/s12889-026-28474-y)
Supplement: Supplementary file 2 [file 12889_2026_28474_MOESM2_ESM.docx]

**Stakeholder Consultation Questionnaire**

**The Intersection Between Maternal Health and Disability Inclusion in Mozambique**

**Introduction:**

Thank you for participating in this consultation. This questionnaire aims to gather insights from key stakeholders on the challenges, gaps, and opportunities related to maternal health services and disability inclusion in Mozambique. Your responses will be invaluable in understanding the current landscape and informing future recommendations.

**Section 1: General Information**

1. Organization:
2. Role/ Position:
3. Years of Experience in Maternal Health and/or Disability Inclusion:

**Section 2: General Perceptions of Maternal Health and Disability Inclusion**

4. How would you describe the current state of maternal health services in Mozambique?
a. Very Satisfactory
b. Satisfactory
c. Neutral
d. Unsatisfactory
e. Very Unsatisfactory
5. To what extent do you believe that women with disabilities have equitable access to maternal health services in Mozambique?
a. Fully Equitable
b. Mostly Equitable
c. Somewhat Equitable
d. Rarely Equitable
e. Not Equitable
6. How well do you think current maternal health services accommodate the needs of women with disabilities?
a. Very well
b. Somewhat well
c. Not well
d. Not at all
e. Don't know
7. In your view, what are the main challenges women with disabilities face in accessing maternal health services in Mozambique?
8. Can you identify any specific areas where maternal health services fail to meet the needs of women with disabilities?
9. What barriers do you think women with disabilities face when trying to access sexual and reproductive health services? (You can select more than one)
a. Physical barriers (e.g., inaccessible facilities)
b. Communication barriers (e.g., lack of sign language interpreters)
c. Attitudinal barriers (e.g., discrimination by healthcare providers)
d. Financial barriers (e.g., cost of services)
e. Other (please specify)
10. How would you assess the availability and adequacy of medical equipment and facilities to accommodate women with disabilities in maternal health settings?
a. Adequate
b. Somewhat Adequate
c. Inadequate

- Comments:

**Section 3: Policy and Practice**

11. Are you aware of any policies or programs in Mozambique that specifically address the needs of women with disabilities in the context of maternal health?
a. Yes (please specify)
b. No
12. In your experience, how effective are these policies or programs in improving access to maternal health services for women with disabilities?
a. Very effective
b. Somewhat effective
c. Not effective
d. Don't know
13. What gaps do you see in the current policies or practices related to maternal health for women with disabilities?
14. What recommendations would you suggest for improving disability inclusion in maternal health services in Mozambique?

**Section 4: Training and Capacity Building**

15. Do you think healthcare providers are adequately trained to handle the specific needs of women with disabilities in maternal health settings?
a. Yes
b. No

- Comments:

16. What kind of training or capacity-building initiatives do you think are necessary for healthcare providers to better serve women with disabilities?
17. How important is it for the voices of women with disabilities to be included in the planning and delivery of maternal health services?
a. Extremely important
b. Very important
c. Somewhat important
d. Not important
e. Don't know

**Section 5: Final Thoughts**

18. Do you have any additional comments or suggestions on how to enhance maternal health services for women with disabilities in Mozambique?

Thank you for your participation!
